# Evaluation of cocoon silk as an annular closure material in a rodent disk model: Minimal behavioral burden despite pronounced inflammatory reaction

**DOI:** 10.1371/journal.pone.0335641

**Published:** 2025-11-18

**Authors:** Friederike Weidemann, Sarah Strauß, Frederik Schlottmann, Mykola Fedchenko, Janin Reifenrath, Dorothea Daentzer

**Affiliations:** 1 Department of Trauma Surgery, Hannover Medical School, Hannover, Germany; 2 Department of Plastic, Aesthetic, Hand and Reconstructive Surgery, Hannover Medical School, Hannover, Germany; 3 Department of Pathology, Hannover Medical School, Hannover, Germany; 4 Hannover Medical School, Department of Orthopaedic Surgery, DIAKOVERE Annastift, Hannover, Germany,; 5 Lower Saxony Center for Biomedical Engineering, Implant Research and Development (NIFE); Emory University School of Medicine, UNITED STATES OF AMERICA

## Abstract

**Background:**

Disk herniation is a common disease in the population. In case of relevant neurologic deficits and/or pain syndrome a surgical approach is necessary. Therefore, an incision has to be made in the outer ring of the disk to remove all parts of the prolapse. To date, a reliable closure device is missing and a recurrent herniation after a pain free interval is a postoperative problem well-known among spine surgeons.

**Methods:**

The current study works on proof of concept and is the first of its kind that discusses cocoon silk as a possible closure material in intervertebral disk defects in a rat model. In addition, the burden of each animal due to the surgical procedure has been evaluated by Von Frey-filament testing and stress evaluation.

**Results:**

The rats represented no or only minor stress response. In the filament testing the animals showed low-grade reactions in general as well. In the examination, inflammatory tissue response was detected directly to the silk, predominantly associated with macrophages. In some areas, cell death was visible.

**Conclusion:**

The stress and pain impact on subjects after silk implantation as an annular closure device in a disk model in rat tails is negligible. The inflammatory reactions might be associated with remaining particles of the spider eggs, not the silk itself. Further investigations would be necessary to overcome this problem.

**Trial registration:**

All animal experimental protocols were approved by the Lower Saxony State Office for Consumer Protection and Food Safety (LAVES; Approval No 33.8-42502-04-21/3771)

## Background

Disk herniation is part of a multifactorial degeneration process which is physiological and begins already in young adults. For the age group between 45 and 55 years, herniated disks are observed in up to 20% in the lumbar spine [1,2]. In case of relevant neurologic deficits and/or pain syndromes that are resistant under conservative treatment, a surgical approach should be offered. The primary aim is the removal of any compressive elements in the spinal canal and neuroforamen to relieve the affected neural structures. Dependent on the underlying morphology of the disk herniation various operative methods exist to eliminate the compromising parts of the disk. Under microscopic or endoscopic view, the currently accepted standard procedure is to remove only loose fragments (sequestrectomy), rather than to radically remove all intersomatic disk material radically (discectomy) [3–6]. However, in all kinds of disk herniation a defect of different size occurs in the outer ring of the disk (annular fibrosus). In some cases, the defect even needs to be expanded to completely remove all parts of the disk prolapse, particularly in chronic situations. To date, a reliable closure device is missing and a recurrent herniation after a pain free interval is a postoperative problem well-known among spine surgeons. The rate is estimated to range from 5 to 25%, and initial defects of ≥ 6 mm are considered to carry a higher risk [7]. In addition to the patient’s individual physical and psychical burden, recurrent disk herniation increases both direct health care costs as well as indirect economic follow-up costs due to prolonged sick leave [8].

Different research approaches have been developed to address this annular defect. A bone-anchored implant (Barricaid®, Intrinsic Therapeutics, Woburn, MA, USA) showed a reduced re-herniation rate compared to single microdiscectomy after a 3-year follow up. However, due to serious handling-related complications, no general recommendation can be made [9]. Furthermore, research is poor with regard to the influence of the Barricaid^®^ bone implant on the vertebrae as well as a debatable bone-disk diffusion impairment of nutrients [10,11]. Currently, the Barricaid^®^ device is no longer available in Europe due to an expired license [12].

Likewise, soft tissue suture devices and fibrin or hydrogel sealant have shown controversial outcomes, and not all have yet reached clinical administration [13–16]. Thus, the clinical need for an annular closure device remains. A potential biomaterial for this purpose could be spider silk.

Numerous studies describe an excellent biocompatibility, which has been demonstrated *in vitro* according to DIN EN ISO 10993–5:2009 and 10993–4:2009 [17,18] and *in vivo* in several rodent [19] and sheep [20,21] models for different applications (e.g. regeneration of peripheral nerves, fasciae replacement, skin healing) as well as in human healing trials [22]. Spidersilk combines high mechanical stability with simultaneous biodegradability [23]. In most published studies, dragline silk (major ampullate silk) has been used, which is harvested as a single thread. In contrast, egg case silk (cocoon or tubuliform silk) is naturally assembled by the spider into a three-dimensional network of interconnected short fibers, which may be advantageous for annular closure [23]. Unlike dragline silk, which mainly consists of major ampullate silk proteins, cocoon silk is a composite material containing both major ampullate and tubuliform gland-derived silks. This structural and biochemical diversity contributes to the distinct mechanical and biological properties of cocoon silk [24]. According to analyses of Naghilou et al. dragline and cocoon silk of *Nephila edulis* (since 2021 listed as *Trichonephila edulis*) have comparable secondary protein structures. The group found both of the silk types suited for seeding of Schwann cells. Data about silk application in intervertebral disks *in vivo* are missing. Therefore, the primary target of the current project was the investigation of cocoon silk of female araneid *Trichonephila edulis* being integrated into damaged intervertebral disks. Cocoon silk is assembled to a three-dimensional network of interconnected short threats by the spider, contrary to the commonly used dragline silk (major ampullate silk), which is harvested as one single thread [25].

As proof of concept, the operation technique was established in the current *in vivo* study and stress evaluation for the animals was verified. The findings of the study did not claim to be able to be transferred to humans.

## Materials and methods

### Spider housing and silk collection

**Fig 1:**
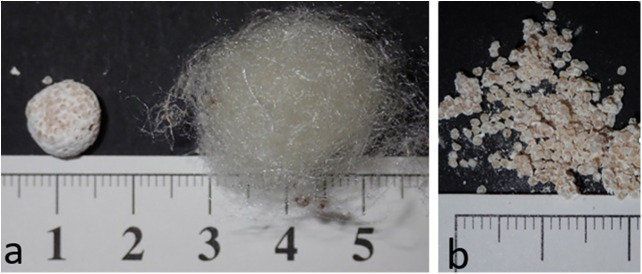
Cocoon silk and egg–particle conglomeration. (a) Cocoon silk and egg-particle conglomeration; overview; (b) egg-particle conglomeration in detail.

*Trichonephila edulis (T. edulis)* spiders were housed as previously described [25]. The animals live free within an own room with temperatures between 20°C (winter) and up to 30°C (summer) and a relative humidity in a range from 40 to 60%. Animals were watered daily with tap water and fed twice a week with crickets (*Acheta domesticus*). Under laboratory conditions *T. edulis* mates throughout the year. Because the spiders are kept freely in the room, targeted mating is not necessary. After fertilization, the female spider leaves her web and builds a cocoon (egg sac), which can contain several hundred eggs. Shortly after completing the cocoon, the animal returns to its web and cocoons can be collected. The eggs, bundled together and coated with particles, were then removed (Fig 1) [26].

Remaining cocoon silk was macroscopically cleaned from visible particles. According to the established standard cleaning procedure, the cocoon silk was thoroughly washed three times in *aqua distillate* by vortexing for 30 seconds each time, then sterilized by autoclaving and dried.

### Animal experiments

All animal experimental protocols were approved by the Lower Saxony State Office for Consumer Protection and Food Safety (LAVES; Approval No 33.8-42502-04-21/3771). The ARRIVE guidelines were followed while reporting. All methods were performed in accordance with relevant guidelines and regulations. Six adult male rats (LEW/HanZtm, Central animal facility, Hannover Medical School, Hanover, Germany) with a body weight of 410.8 g + /- 8.3 g were included in the study. The rats were housed in pairs (cage type IVs, Uno Roestvaststaal BV, Netherlands) at room temperature between 20 and 24°C, relative humidity of 45–65% and 14/10 h day–night cycle with free access to standard diet (1324 TPF, Altromin, Lage/Westphalia, Germany) and water. Animals were handled several times per week for five weeks to adapt them to the personnel and their surroundings.

### Anesthesia and analgesia

Surgery was performed under isoflurane anesthesia (2–5 vol% in Oxygen; Isofluran CP, cp-pharma, Burgdorf, Germany) and additional local anesthesia at the tail base (10 mg/kg Lidocainhydrochlorid 2%, s.c., bela-pharm, Vechta, Germany). The rats received carprofen (5 mg/kg s.c., Rimadyl^®^, Zoetis, Berlin, Germany) 30 minutes and buprenorphine (0.05 mg/kg s.c., Bupresol^®^, vet, cp-Pharma, Burgdorf, Germany) 15–20 minutes before the operation as pre-emptive pain medications and an additional subcutaneous postoperative buprenorphine injection approximately 4 hours after surgery.

The administration of carprofen was continued for five days postoperatively. Carprofen could have been given for extended periods to manage pain as further treatment option.

Enrofloxacin (5 mg/kg, s.c., Baytril^®^, Bayer, Leverkusen, Germany) was applied preoperatively and four days after surgery as antibiotic prophylaxis.

### Surgical procedure

**Fig 2:**
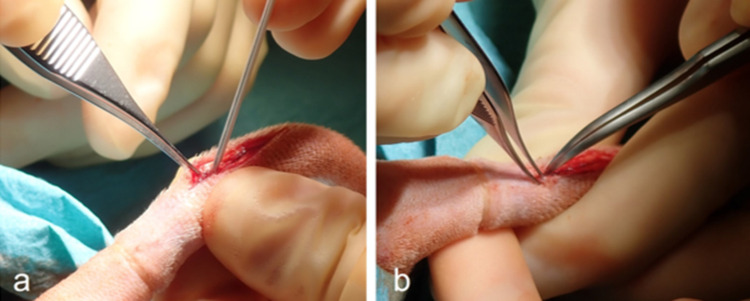
Surgical procedure: (a) Stabbing in the intervertebral space with an 18 G cannula; (b) placing the silk with pointed tweezers.

The hairy tail base, as well as the remaining portion of the rat tail, was shaved and disinfected with povidone-iodine solution. To minimize possible bleeding during surgery, an elastic band was placed at the tail base. The rat was then positioned prone on a warmed pad. The tail was covered with sterile drapes, and a second careful disinfection was performed. A dorsal skin incision was made, approximately 3 cm in length, at the level of coccygeal vertebrae (Co) 4−5 to Co 5−6. These segments were chosen for practical reasons. The vertebral level of interest was identified by palpation. The soft tissue was gently prepared down to the disk area. To create a disk defect, an 18 G cannula was inserted into the intervertebral space. The defect was applied to two adjacent discs. One defect was left empty (blank sample), while the other was filled with a small pellet of spider silk. The amount of silk was visually estimated and kept comparable between animals. Bleeding, if necessary, was controlled by compression. Wound closure was performed in single stitch technique with a resorbable suture (Vicryl 4−0, Ethicon, Johnson&Johnson, Raritan, NJ, USA) (Fig 2).

### Stress evaluation

The animals were observed for six weeks overall after surgery to assess the stress level of the surgery and monitor the postoperative regime. Daily check-ups were accomplished during the first two weeks (Table 1). Depending on the tension level further controls were performed daily or reduced to three times weekly. The overall burden corresponded to the highest level within a subcategory. Stress levels and subsequent measurements were recorded on a rat-specific score sheet adapted to the experimental procedure (Table 2). The assessment was based on the Directive 2010/63/EU of the European Parliament and the guidelines of the Working Group of Berlin Animal Officers. The official authorization was obtained from LAVES.

**Table 1:** Score sheet for the postoperative evaluation of general condition, behavior and surgical wound.

score value	body weight	general condition	behavior	surgical wound
1	• weight gain • steady • weight loss ≤ 9.9%	• coat smooth, shiny, orifices clean • defecation and urine secretion normal • eyes bright	• awake, attentive, curious • physiological body posture and gait	• no secretion • no swelling • no hyperthermia • no redness • no dehiscence • tail movement normal
2	• weight loss 10-14.9%	• coat matted focally • coat defect • defecation and urine secretion normal	• calm, attentive, allotriophagy (pica-behavior) • reduced movement • reduced cleaning behavior • species specific body posture	• possible secretion • possible swelling • possible hyperthermia • possible redness • possible • tail movement possibly reduced
3	• weight loss 15-19.9%	• coat matted, shaggy • neglected orifices, slightly affected	• very calm • inattentive • weak • sternoabdominal posture • reduced motion	• possible secretion • possible swelling • hyperthermia < 24 h • possible redness • possible • tail movement possibly reduced
4	• weight loss ≥ 20%	• dirty coat • sticky or moist orifices • defecation and urine secretion impaired • atypical body posture • eyes sticky and sunken	• apathetic, lateral posture • pronounced hyperkinetic • stereotypes of behavior • coordination disorders • automutilation	• possible secretion with pus • possible swelling • hyperthermia < 24 h • possible redness • dehiscence • tail movement reduced

**Table 2:** Score sheet for stress evaluation and assigned measures.

Score value	Assessment	Measures
1	no stress	• none
2	minor stress	• careful observation (at least once a day) • adjuvant therapy (infrared heat lamp, pain medication, soaked food)
3	moderate stress	• careful observation (at least once a day) • careful observation (twice a day) on the occurrence of social disorder/decreased general health • adjuvant therapy (infusion, pain medication and/or antibiotic treatment) • symptoms > 48 h: euthanasia
4	intense stress	• euthanasia

The behavior of each rat was observed for at least 5 minutes. To evaluate the movement of the tail properly, the rat was placed on a table to run free. In addition, the determination of mechanical pain was performed using Von Frey-filaments [27,28]. Testing was performed two days preoperatively and on postoperative days 2, 7, 14, 21, 28 and 42. For each test, the rat was placed on a lattice, allowed to acclimate for 5 minutes, and then stimulated on the ventral tail with Von Frey-filaments (Marstock Nerve test, Schriesheim, Germany) with strength ranging from 2 g (19.6 mN) to 0.5 g (4.9 mN) for six seconds each. The filament was applied until it bent slightly, and five repetitions were performed with each filament strength.

If the rat avoided the filament, shook off the base of the tail or licked itself within six seconds after the filament stimulus, the response was evaluated as “positive”. If there was none of the reactions mentioned, the response was evaluated as “missing”.

The Von Frey-filament test was continued in descending filament strength until no further “positive” reactions were observed.

### Histological preparation

Six weeks after surgery, the animals were anesthetized and sacrificed. Vertebrae were sectioned using a diamond saw (EXAKT Advanced Technologies GmbH, Norderstedt, Germany), and the target areas (Co 4–5 and Co 5–6) were prepared. Adjacent areas Co 3–4 and Co 6–7 were used as healthy controls.

Samples were fixed in 4% buffered formalin (Medite Medical GmbH, Burgdorf, Germany) for at least two days at 4°C and embedded in methyl methacrylate (Technovit^®^ 9100 New, Heraeus-Kulzer, Hanau, Germany) for histological analyses according to the manufacturer’s instructions and established protocols [29].

4 µm thin longitudinal sections were cut with a microtome (RM 2155, Leica, Bensheim, Germany) and associated tungsten carbide knives (Leica, Bensheim, Germany), placed on poly-L-lysine coated glass slides, stretched with 90% ethanol, covered with a polyethylene foil, pressed and dried for at least 48 h at 37°C. For Toluidine blue staining (0.1%; Sigma-Aldrich Chemie, Taufkirchen, Germany, 30 sec), sections were deacrylated in xylene (2 × 10 min) and 2-methoxyethylacetate (1 × 10 min) and rehydrated with a decreasing alcohol series. Afterwards, slices were washed with distilled water, dehydrated in an increasing alcohol series, and mounted with Eukitt^®^ (Labonord, Mönchengladbach, Germany). After drying at room temperature for at least 24 h light microscopic examination was performed with an Axioskop 40 microscope supplemented by a polarization optics to countercheck for silk detection. Images were taken with an AxioCam Mrc digital camera and associated Axio Vision Software (Carl Zeiss, Oberkochen Germany). Evaluation was performed descriptively.

## Results

### Stress evaluation

During the observation period the animals reached no or minor stress levels in the overall burden. For all rats the weight loss was always ≤ 9.9%, minor stress levels were detected by “general condition”, “behavior” and “surgical wound”. Rat #1 and rat #4 showed in all sub-categories score levels of one. Rat #3 exhibited a score level of two in general condition on a single occasion. For sub-category “behavior” rat #5 reached the minor stress level twice and rat #6 three times. These three subjects showed increased stress levels within the first three postoperative days.

**Fig 3:**
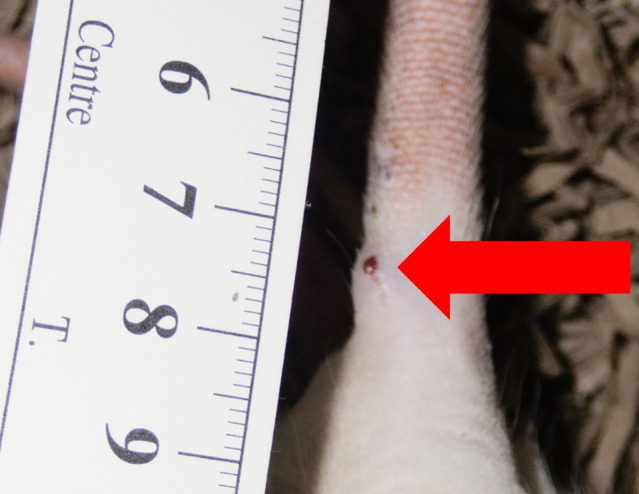
Tail of rat #2 at level Co 4-5: Soft tissue growth, overview, scale is displayed in cm.

Rat #2 developed a subcutaneous circumferential growth on the 16^th^ postoperative day, which did not affect the animal’s behavior or the general condition, and no pain could be induced due to palpation (Fig 3). After nine days, no further changes or growth could be observed.

In the filament testing, the animals generally showed low-grade reactions. Preoperatively, one rat showed once a reaction during the 6.9 mN filament test. Postoperatively, two subjects responded to stimulation with the 19.6 mN filament: rat #1 in three out of five repetitions, and rat #2 in one. During the subsequently filament (13.7 mN) test, only rat #1 showed a response, manifested as grooming of the stimulated area and tail lifting. No responses were detected in any animal during the other filament tests.

In summary, the rats showed only slight signs of discomfort for up to two days postoperatively, with no further observable behaviors indicative of discomfort or pain.

### Histological examination

The *post-mortem* dissection of the operated vertebrae revealed that the target area at the level Co 4–5 to Co 5–6 was not always accurately selected by palpation. In rats #3 to #5 the defects were created in Co 5–6 and Co 6–7, while in rat #6 the defects were placed one position higher than planned (Co 3–4 and Co 4–5). For the histological examination, vertebrae levels Co 3–4 and Co 6–7 were prepared and additionally analyzed in all animals as unaffected controls. Therefore, no data were missing, and the results showed no impact regarding the outcome of the prepared segment.

**Fig 4:**
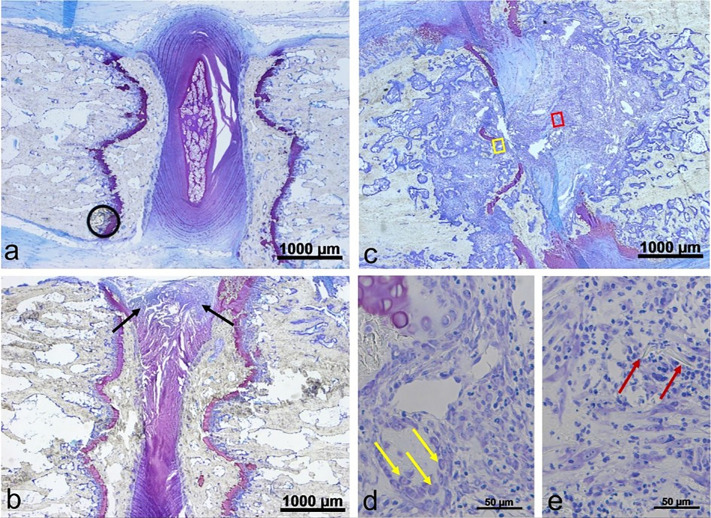
Histological analysis of rat intervertebral discs after silk implantation. (a) Intervertebral disk of the rat: Unprocessed specimen with nucleus pulposus and fibrocartilage between the vertebral bodies; (b) intervertebral space after needle pucture without silk implantation showing slight degenerative changes (black arrows); (c) resorptive inflammatory reaction involving of the adjacent vertebral bodies six weeks after silk implementation in the intervertebral disc; (d) and (e) magnified view of the inflammatory area showing osteoclasts around remaining osseous structures (d, yellow arrows) and residual silk material (e, red arrows); staining Toluidine blue.

The histological examination of the disks filled with silk showed high grade inflammatory changes and foreign-body reaction. The inflammation was directly associated with the silk material. Predominantly, a macrophage associated resorptive inflammation could be observed. Foreign body giant cells could be detected around residual silk material. In some areas, cell death was visible. Granulocytes were seen only in a minor content. In some preserved specimen the bone area showed similar reactions towards the silk contact. The epiphysis was completely resolved and inflammatory bone reactions were detected (Fig 4).

In two animals (rat #2 and #3) the silk filling was found in the surrounding tissue or at the transition from disk to soft tissue. It remains unclear, if the silk was placed outside the target area during surgery or displaced during the postoperative follow-up. Histological examination of the wound defect of rat #2 was additionally performed. It revealed characteristics of granuloma and contained silk residuals. Disks stabbed with the cannula but left without silk filling showed no inflammatory changes, only slight degenerative alterations.

**Fig 5:**
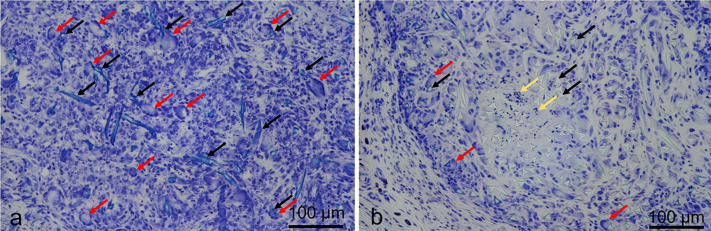
Inflammatory tissue response to silk material in rat tail intervertebral region. (a) Inflammatory tissue response in the rat tail: residual silk material (black arrows) in rat #3 (transition from disk to peri-discal soft tissue) is surrounded by inflammatory cells, predominantly macrophages, merged to foreign body giant cells (red arrows); (b) rat #2, the silk was displaced in peri-discal soft tissue (see Fig 3 for clinical finding) and developed a granuloma with similar cell content. Cellular fragments were visible (yellow arrows) in-between silk fibres; staining Toluidine blue.

Regardless of the surrounding tissue, the silk caused an inflammatory reaction (Fig 5).

## Discussion

Inducing structural disk degeneration by needle puncture is a well-established model in rodents, although information about pain and behavioral changes of the animals have rarely been documented [30,31]. It should be noted that needle puncture itself is more likely to induce spontaneous healing than in progressive long-term degeneration. As proof of concept, the current study investigated the influence of an intradiscal silk implant after needle puncture on the rats’ stress level.

Findings regarding stress evaluation fulfilled expectations. In summary, the animals reached no or minor stress levels in the scoring and showed low defense reaction in the Von Frey filament testing. No animal reached moderate scored stress levels, humane endpoints or died before sacrifice.

A comparison with the work of Lai et al. offers further context; however, methodological differences need to be taken into account [31]. In their model, pain behavior was assessed by paw withdrawal thresholds following intradiscal injection of TNFα, which reliably induced mechanical hypersensitivity. In contrast, our study evaluated stress and pain-related responses using Von Frey stimulation of the ventral tail after needle puncture. Given that pain sensitivity can vary across tissues, the results are not directly comparable. Nevertheless, both studies emphasize the link between local inflammatory processes within the intervertebral disc and behavioral correlates of pain. Notably, whereas TNFα injection in Lai’s model produced clear hypersensitivity, the pronounced inflammatory response surrounding the silk implant in our animals did not result in detectable increases in pain-related behavior.

Recent work by Wang et al. (2023) highlighted that microfractures of the endplate can initiate a cascade of changes, including Modic-like alterations, intervertebral disc degeneration, and spinal cord sensitization [32]. These results point to a mechanistic link between structural damage at the endplate and neuroinflammatory responses, underscoring the view that the disc, endplates, and adjacent vertebrae form an interdependent unit relevant for pain generation.

Taken these studies together, our findings (Fig 4C) extend these observations by showing that pronounced local inflammation following silk implantation did not lead to behavioral sensitization. This contrast suggests that, while structural endplate disruption may facilitate the translation of inflammation into pain-related outcomes, inflammation alone is insufficient. Thus, the broader tissue context—particularly the interaction between disc, endplate, and vertebral bone—appears to be a critical determinant in shaping pain phenotypes.

Wawrose et al. established a rat model to investigate intervertebral disk degeneration and pain-related behavior [33]. Disk degeneration was simulated by percutaneous annular puncture. Manner was documented by video camera and assessed by Ethovision XT Automated Behavior Recognition software. The parameters of animal motion were selected to one representative parameter which referred to low back pain. Significant differences were detected in total distance movement, supported and unsupported rearing duration as well as in the twitching frequency. The results were interpreted as an increase of pain related behavior. Besides, they measured RANTES, a proinflammatory cytokine in the serum. Interestingly RANTES concentration in the experimental group was similar to an unpunctured control group and decreased during observation period.

The influence to pain related behavior due to inflammatory reaction is still discussed controversially in the model of intervertebral disk injury [27,28]. Nevertheless, the recent study displayed significantly high inflammatory response towards spider silk with low measured pain or stress levels. Compared to the preoperative status the animals showed no changes of behavior when running freely on a table. While Carprofen was administered for five days postoperatively, its anti-inflammatory effect could be a reason for the current results.

**Fig 6:**
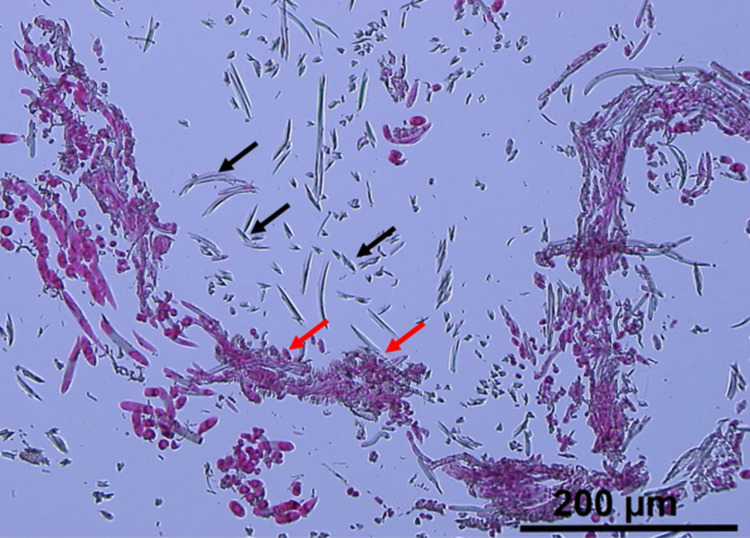
Remaining particles (red arrows) in between native cocoon silk (black arrows); haematoxylin-eosin.

Granuloma formation in context of implantation of *Trichonephila* (golden silk orb weaver) spider silk has been described rarely. Vollrath et al. reported granuloma formation with appearance of giant cells as reaction on subcutaneous implantation of *Trichonephila clavipes* (former named *Nephila clavipes*) dragline silk in a pig model [34]. Koop et al. implanted a combination of *T. edulis* dragline silk and fibrin in the spinal cord of rats [35]. All animals showed massive granuloma formation as response. In many other animal studies in rat and sheep [19,21,36,37] as well as healing trials in humans [22] no such massive reaction has been described. Koop et al. hypothesized that the reaction might occur in context of the specific immunology of the central nervous system, which is different from the skin, muscle and peripheral nervous system [35]. All of the mentioned studies used dragline silk, while in our study cocoon silk was chosen due to the three-dimensional interconnected fiber network which seems to be more useful than single threats of silk. As already mentioned in the material and methods section, the spider eggs are coated with particles which we tried to remove by washing the silk [26]. An incomplete removal and therefore remnants might be the cause for the massive granuloma formation *in vivo*. The results of a project using cocoon silk as matrix for tissue engineering, which was performed in parallel to the recent study, support this consideration as histological analyses clearly showed remaining particles (Fig 6) [38].

In contrast to the *in vivo* application, particles did not develop any negative influence on cells (keratinocytes, adipose derived stromal cells, dental pulpa stem cells) *in vitro* [38]. Even very sensitive cells like Schwann cells can be grown on cocoon silk and display no differences in vitality or morphology compared to cells seeded on dragline silk [39]. Further support of our hypothesis might be that amino acid profiles of major ampullate (dragline) and cylindrical gland (cocoon silk) is very similar in *T. edulis’* close relative *T. clavata* (former named *Nephila clavata*) [40]. Therefore, it is unlikely that reactions are caused by the silk itself.

Gellynck et al. reported severe inflammatory response followed by fibrosis on egg case silk of *Araneus diadematus* (European garden spider) subcutaneously implanted in Wistar rats [41]. These reactions could be reduced by enzymatical pre-treatment of the silk, but this resulted in altered mechanical characteristics, e.g. reduction of elasticity and breaking strength. The used enzymes, proteinase K and trypsin, were able to reduce or remove protein-based debris as well as protein layers on the silk, or even broke up silk’s score structure. Unfortunately, there was no information whether a control was treated with phosphate buffered saline and received the same additional washing steps. Furthermore, the authors did not report whether the silk was examined visually or histologically for contamination with any kind of residues from the egg batches. Therefore, the question of the cause of the severe reaction cannot yet be answered. As a next step in our project a further detailed *in vitro* evaluation with cell lines, supporting inflammatory reactions, will be performed analyzing the inflammatory potential of native sterilized and pretreated *T. edulis* cocoon silk.

## Conclusion

The stress and pain impact on subjects after silk implantation as an annular closure device in a disk model in rat tails is negligible. Therefore, basically the model is suitable for further studies. Problems associated with placing and fixation of silk in the disk should be addressed in future studies. Furthermore, suitable imaging and fixing techniques need to be evaluated.

However, severe inflammatory reaction towards *T. edulis* cocoon silk was detected. The precise cause could not be specified in the present study, whereas in our opinion the silk itself unlikely seems to be the reason. Remaining particles on the silk have to be discussed more intensively and further studies are necessary to get clarification in this field of research.
